# MonoSeg: An Infrared UAV Perspective Vehicle Instance Segmentation Model with Strong Adaptability and Integrity

**DOI:** 10.3390/s25010225

**Published:** 2025-01-03

**Authors:** Peng Huang, Yan Yin, Kaifeng Hu, Weidong Yang

**Affiliations:** National Key Laboratory of Multispectral Information Intelligent Processing Technology, School of Artificial Intelligence and Automation, Huazhong University of Science and Technology, Wuhan 430000, China; penghuang@hust.edu.cn (P.H.); yanyin@hust.edu.cn (Y.Y.); hukaifeng@hust.edu.cn (K.H.)

**Keywords:** UAV perspective, infrared image target recognition, instance segmentation, low computation

## Abstract

Despite rapid progress in UAV-based infrared vehicle detection, achieving reliable target recognition remains challenging due to dynamic viewpoint variations and platform instability. The inherent limitations of infrared imaging, particularly low contrast ratios and thermal crossover effects, significantly compromise detection accuracy. Moreover, the computational constraints of edge computing platforms pose a fundamental challenge in balancing real-time processing requirements with detection performance. Here, we present MonoSeg, a novel instance segmentation framework optimized for UAV perspective infrared vehicle detection. Our approach introduces three key innovations: (1) the Ghost Feature Bottle Cross module (GFBC), which enhances backbone feature extraction efficiency while significantly reducing computational over-head; (2) the Scale Feature Recombination module (SFR), which optimizes feature selection in the Neck stage through adaptive multi-scale fusion; and (3) Comprehensive Loss function that enforces precise instance boundary delineation. Extensive experimental evaluation on bench-mark datasets demonstrates that MonoSeg achieves state-of-the-art performance across standard metrics, including Box mAP and Mask mAP, while maintaining substantially lower computational requirements compared to existing methods.

## 1. Introduction

With the maturation of unmanned aerial vehicle (UAV) technology, it has emerged as a new platform for visual perception, demonstrating significant value across various fields such as transportation, mapping, agriculture, military operations, and rescue missions [1,2]. By integrating infrared imaging technology, which offers visibility under low-light conditions and the capability to generate images based on thermal radiation, UAVs can undertake more complex tasks [3,4], including intelligent agricultural management, traffic surveillance, and fire safety, thereby greatly expanding their application scope and potential. The fusion of infrared imagery with advanced deep learning algorithms equips UAVs with the ability to identify targets in all weather conditions at medium to low altitudes [5,6]. This integration not only enhances overall capabilities in reconnaissance, monitoring, and search but also specifically improves the efficiency of identifying ground vehicle targets. However, more granular recognition methods, such as instance segmentation, have yet to be widely adopted in UAV systems.

Instance segmentation algorithms have found extensive applications in common object recognition, medical image processing, and general object recognition with large models, demonstrating their fine-grained pixel classification capabilities and significant advantages in accurately identifying objects [7]. However, in the current domain of target recognition from a UAV perspective, the most commonly employed approach remains object detection algorithms. Despite the potential of instance segmentation, it has not yet been widely adopted in scenarios involving both monomodal visible light and bimodal infrared imaging.

The instance segmentation algorithm has been shown to be a more refined method than the object detection algorithm [8,9], which improves the ability of infrared UAV perspective recognition of vehicle targets. These algorithms distinguish between targets and backgrounds at the pixel level based on semantic information, thus offering superior target recognition performance. However, instance segmentation of infrared vehicle targets from a UAV perspective encounters numerous challenges:

1. Infrared imaging suffers from low resolution and limited information content, and the contrast between vehicle targets and the background is weak, with blurred edges, making it difficult to distinguish between them. The thermal signatures of targets may be incomplete, and the thermal radiation levels of vehicle targets and background areas can be homogeneous, further complicating differentiation.
2. When infrared imaging is integrated into airborne scenarios, the degree of freedom in imaging significantly increases. Factors such as the UAV’s varying flight altitudes, infrared imaging angles, imaging times, and the choice between long-wave and short-wave imaging methods all impact the final image quality, thereby increasing the difficulty of segmentation and recognition.
3. As lightweight platforms, UAVs have limited payload capacities, which restricts the computational power of the AI processing units they can carry. Therefore, algorithm design must prioritize high precision while striving to minimize computational demands.
4. Currently, there is a scarcity of datasets for UAV perspective infrared image segmentation, and datasets specifically targeting vehicle segmentation in airborne infrared images are entirely lacking. Consequently, for this task, it is necessary to construct a new dataset to support algorithm development and training.

To address these issues, we constructed a drone-perspective infrared vehicle target instance segmentation dataset, DIVIS (Drone Infrared Vehicle Instance Segmentation dataset), utilizing the SAM large model to assist in annotation [10]. We also improved the multi-scale adaptability and segmentation integrity of the YOLOv8-Seg baseline algorithm [11,12], achieving superior recognition performance. Furthermore, the model’s speed has been significantly enhanced, which is essential for rapid in-flight decision-making by high-speed UAVs.

The baseline model exhibits limitations in scale adaptability. As the model depth increases, the rise in information entropy does not translate into efficient feature propagation [13]. To address this issue, we propose the Ghost Feature Bottle Cross module (GFBC). This is an improvement module designed for the feature extraction backbone of the model. It combines Ghost features with the Bottle Cross structure [14,15], enhancing the model’s multi-level receptive field hierarchy and improving its perception of targets.

During the feature integration phase in the model neck layers, information loss can easily occur when data is passed through the second group of feature pyramids [16], primarily due to information loss during changes in feature sampling dimensions. To address this issue, we propose the Scale Feature Recombination module (SFR). This module utilizes dual-scale convolutions along with channel shuffling in high-density information layers [17], which enhances the information utilization rate of the neck layers.

In the pixel-level classification part of the final head, we address the issue of low segmentation mask coverage in the target edge regions. Building upon the Region BCE Loss used in the baseline method [18], we propose the target Mask Weighted BCE Loss and the Mask Dice Loss [19]. The Target Mask Weighted BCE Loss aims to balance the positive and negative sample distribution, while the Mask Dice Loss focuses on the overall convergence of positive samples towards the ground truth. The combination of these multiple losses improves the pixel classification accuracy of the final output.

To address the challenges in the field of infrared vehicle target instance segmentation from a UAV perspective, our contributions are as follows:

1. We constructed an instance segmentation dataset from a UAV perspective, comprising over 3300 infrared images that include two categories of targets. The dataset features multiple viewpoints, altitudes, and both long-wave and short-wave characteristics.
2. We developed a high-precision instance segmentation algorithm, introducing the Ghost Feature Bottle Cross module (GFBC) and the Scale Feature Recombination module (SFR), along with corresponding improvements to segmentation loss functions.
3. Our algorithm maintains high accuracy while keeping the computational load low, thereby achieving real-time performance that meets the requirements of embedded applications.

The remainder of this paper is organized as follows: Section 2 reviews the related work on instance segmentation algorithms. Section 3 details the design of our proposed DIVIS dataset. Section 4 introduces the specifics of the MonoSeg instance segmentation algorithm. Section 5 presents a comparative performance analysis with similar algorithms and provides an extensive analysis of the experimental results. Section 6 discusses the strengths and weaknesses of the MonoSeg algorithm, along with potential directions for future development. Finally, Section 7 summarizes the key findings and contributions of this research.

## 2. Related Works

Although traditional segmentation algorithms still retain certain advantages in specific domains, the drawbacks of non-deep learning algorithms have become increasingly evident since the emergence of image classification algorithms. Classic algorithms in the field of image segmentation, such as thresholding, edge gradient segmentation, and region growing, are no longer suitable for the high-performance requirements of modern applications. Consequently, the adoption of deep learning-based algorithms has grown significantly, and almost every mention of instance segmentation algorithms is in the context of deep learning approaches [20].

Based on computational requirements and average performance, instance segmentation algorithms can be categorized into lightweight and heavyweight (or real-time and non-real-time) instance segmentation algorithms [20]. Lightweight instance segmentation algorithms are characterized by low computational demands, fewer model parameters, and smaller model sizes, making them suitable for rapid training and deployment. Heavyweight instance segmentation algorithms, on the other hand, are marked by a large number of model parameters and high computational demands, offering significantly superior segmentation and recognition performance compared to standard models and even supporting multi-modal tasks.

### 2.1. Lightweight Instance Segmentation Algorithm

DeepMask [21] is an early algorithm used for object instance segmentation, replacing the approach that combined Box-based region recognition with seed point segmentation and superpixel fusion. It achieved pure deep learning instance segmentation through scale-reduced Mask prediction. Mask R-CNN [18] is a typical two-stage model that introduces a fully connected Mask branch for segmentation, building upon the Faster R-CNN [22] framework. IASS [23] adopts a similar design philosophy, sequentially outputting Class, Box, and Mask through three hierarchical stages.

CondInst [24] introduced an instance segmentation method that does not require ROI (Region of Interest) cropping, utilizing the upper and lower layers of a pyramid structure to output bounding boxes and masks, respectively. However, it still relies on FCN [25] for mask generation, which can impose a certain computational burden on the model. Building upon CondInst, BoxInst [26] presents a weak supervised instance segmentation approach that does not require mask annotations [27]. It uses only bounding boxes as supervisory information, aggregating pixels with strong consistency within the region to form self-learned masks.

SOLO [28] and SOLOv2 [29] employ a feature map grid for target center localization, classification, and instance segmentation, using two parallel branches to constrain the category and mask of each object within the grid. RTMSeg is an extension of the RTMDet algorithm [30], featuring a backbone built with small-kernel dense convolutions and depthwise separable convolutions, and it merges outputs through upsampling from three output heads. YOLACT [31] pioneered the concept of a fully single-stage segmentation model by introducing the idea of prototype masks, which constructs one-to-one box and mask segmentations, thereby achieving instance segmentation with extremely low computational overhead.

YOLO-Seg draws inspiration from the prototype mask concept, combining the high accuracy and low computational load characteristics of successive YOLO detection algorithms [32]. It constructs an instance segmentation algorithm by coupling a target detection head with a prototype mask instance segmentation head. Over time, multiple generations of YOLO-Seg algorithms have been developed, incorporating improvements such as Mosaic data augmentation, CSP (Cross Stage Partial) high-performance modules, decoupled feature heads, and parallel spatial attention modules. These enhancements make YOLO-Seg a preferred algorithm under low-computational-power conditions.

The aforementioned algorithms both fall into the category of single-stage lightweight algorithms; however, they exhibit varying levels of accuracy and speed. In UAV systems, achieving a balance between high-speed decision-making and accuracy is critical. Consequently, subsequent algorithm design has drawn upon the superior design principles of these approaches, with a focused effort on enhancing both speed and precision.

### 2.2. Heavyweight Instance Segmentation Algorithm

Mask2Former [33] is an improved segmentation version of DETR [34], which uses a set of fixed object queries as input for the query side, decoding to obtain the classification output of a group of masks. SOLQ [35] draws inspiration from the expansion approach from Faster R-CNN to Mask R-CNN, proposing the UQR structure based on DETR. This structure connects the query vectors to two MLP (Multi-Layer Perceptron) branches, achieving a unified output of mask vectors and segmentation results. MaskCLIP [36] integrates instance segmentation with CLIP, using word embedding vectors to filter image features, enabling the model to perform dense segmentation on unknown objects based on unfamiliar words.

SAM [10] pioneers the technique of multi-prompt segmentation. Drawing on the prompt strategy from the Natural Language Processing (NLP) domain, SAM supports various types of prior information as input. It compiles the inputs into prompt encodings, which are then combined with positional encoding weights. This process feeds query information into the high-dimensional feature space, generating a set of candidate segmentation results that match the features. These candidates are subsequently scored and filtered using a Two-Way MLP.

In the subsequent dataset construction phase, this paper employs heavyweight instance segmentation algorithms. However, the engineering algorithms deployed on UAVs primarily consist of lightweight algorithms.

## 3. Dataset

Given the severe lack of instance segmentation datasets for infrared vehicle targets from a UAV perspective, and considering that there are existing datasets of a considerable size for vehicle detection in UAV infrared scenes, this paper utilizes these image datasets. By employing segmentation annotations, we have constructed an instance segmentation dataset.

**Figure 1:**
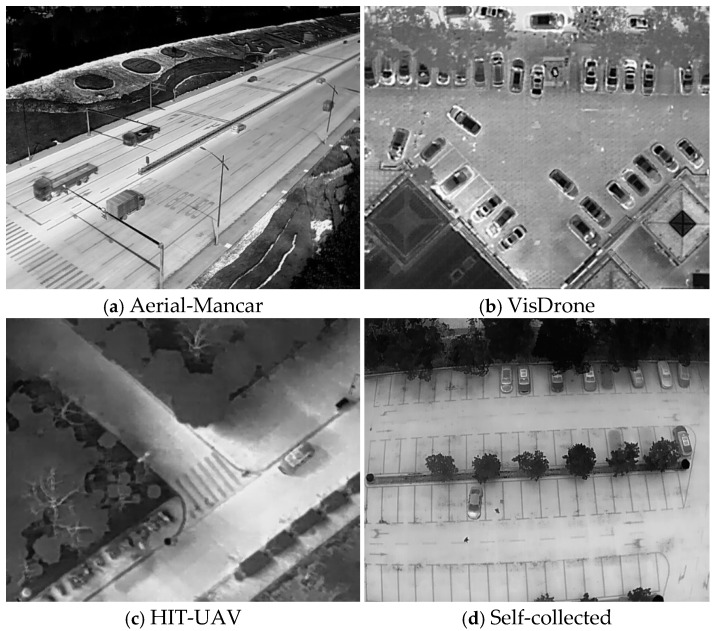
Examples from the four image datasets: (**a**–**c**) publicly available datasets and (**d**) data we gathered ourselves.

Our dataset is constructed from four image collections: Aerial-Mancar [37], VisDrone [38], HIT-UAV [39], and self-collected infrared images from our own UAV, as shown in Figure 1. Each dataset captures scenes with different preferences, aiming to cover a wide range of UAV infrared camera imaging altitudes, angles, and even wavelength types. Therefore, we decided to combine these images to create a comprehensive dataset.

Each of these image sets contains several thousand to tens of thousands of samples. However, since they are sequential data with many repetitive patterns, using all frames could lead to overfitting during model training. Additionally, to ensure a balanced number of samples from each dataset, we performed frame sampling and integration within each image set. The properties of the images are shown in Table 1.

**Figure 2:**
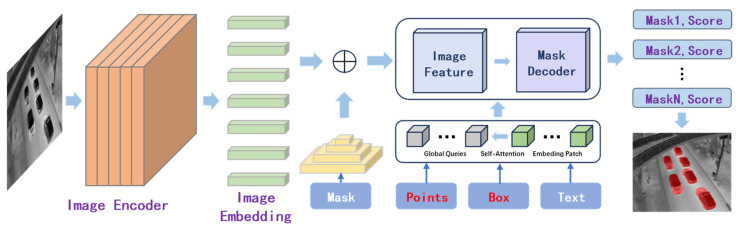
Illustration of SAM large model-assisted annotation [10].

After composing the image dataset, we utilized the SAM model [10] for image annotation. As shown in Figure 2, leveraging the detection box annotations and keypoint information provided in some of the original image datasets, we performed initial labeling and subsequent manual corrections. This process allowed us to build an initial dataset. We then trained a base model using instance segmentation algorithms and used it to predict labels for images with no prior annotations, such as those in the self-collected dataset. Following this, we conducted further manual corrections to finalize the labeling of all images.

Ultimately, we constructed a preliminary drone-perspective infrared vehicle target segmentation dataset—DIVIS (Drone Infrared Vehicle Instance Segmentation dataset). This dataset includes 3303 single-channel infrared images, each with a resolution of 640 × 512. The targets in the images are divided into two categories: small_vehicle and large_vehicle. The small_vehicle category contains 33,685 instances, including sedans, small vans, minivans, and pickup trucks. The large_vehicle category contains 2256 instances, including large trucks, heavy-duty trucks, buses, trailers, and construction vehicles.

**Figure 3:**
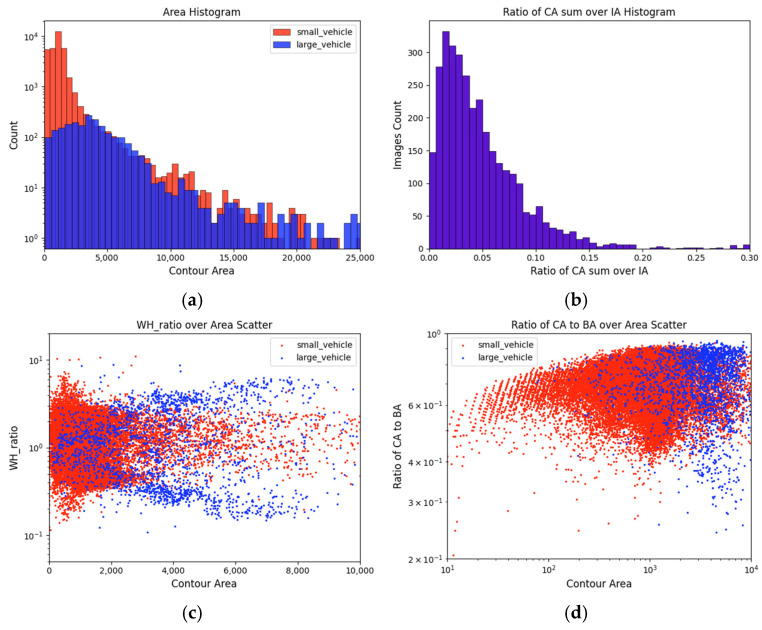
DIVIS dataset statistical properties. (**a**) The histogram of the area distribution of all instances. (**b**) The histogram of the ratio of the sum of contour areas to the image area. (**c**) A scatter plot of the aspect ratio of instance bounding boxes versus the ratio of instance contour area. (**d**) A scatter plot of the ratio of instance contour area to instance bounding box area against the instance area.

As shown in Figure 3, the two categories of targets in the DIVIS dataset exhibit significant differences in terms of size, area, and aspect ratio. The feature distributions of small_vehicle and large_vehicle tend towards two extremes. Small-sized targets have shapes that are more balanced relative to their bounding boxes, whereas, as the area increases, the aspect ratios of the targets deviate more from the central values. The distinct feature distributions of the two target types present certain challenges for algorithm design, requiring the algorithm to handle both target scales and segmentation integrity while maintaining a low computational cost.

## 4. Method

### 4.1. Architecture Overview

This paper addresses scenarios with low computational power, where targets are extremely distributed and highly variable. To balance speed and accuracy, we selected YOLOv8n-Seg [11] as the baseline model. This model offers low computational requirements and high precision across multiple scenarios, and it inherits the robust performance of the YOLOv5 [40] in embedded engineering applications. Given the low contrast between targets and backgrounds in infrared images and the resulting low target recognizability, it is necessary to enhance the segmentation model’s understanding of pixel-level semantics. Therefore, our improvements to the baseline model focus on three aspects: backbone feature extraction, information integration and filtering, and pixel-level prediction loss.

**Figure 4:**
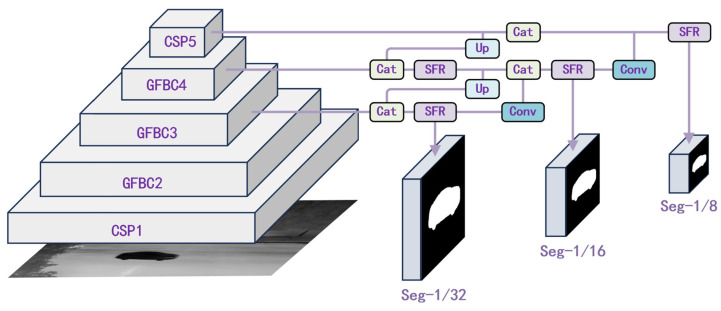
Architecture diagram of the MonoSeg model.

To achieve these objectives, we propose an improved instance segmentation algorithm called Multi-dimension Objects Nano Oriented Segmentation (MonoSeg), as shown in Figure 4. This algorithm possesses strong adaptability to target features and reinforces semantic prediction at the nano-pixel level. It effectively enhances the performance of infrared vehicle target recognition in varied UAV perspectives.

We introduce the Ghost Feature Bottle Cross module (GFBC) in the backbone feature extraction part of the five-level pyramid structure to enhance the information density captured by the model. As the pyramid layers gradually contract, the information entropy in the convolutional layers determines whether each target is retained. The top three layers of the backbone are progressively connected to the neck part of the model. The Scale Feature Recombination module (SFR) filters target and background information, integrating features at two different scales and guiding the model to select the most beneficial information across channels. Subsequently, the three outputs from the neck layer are directed to the head part. Thanks to the prototype design, the head layer can directly output the corresponding mask for each target based on its prediction vector. The three-level predictions accommodate targets of different scales, ensuring robust performance across all scenarios.

### 4.2. Ghost Feature Bottle Cross Module

**Figure 5:**
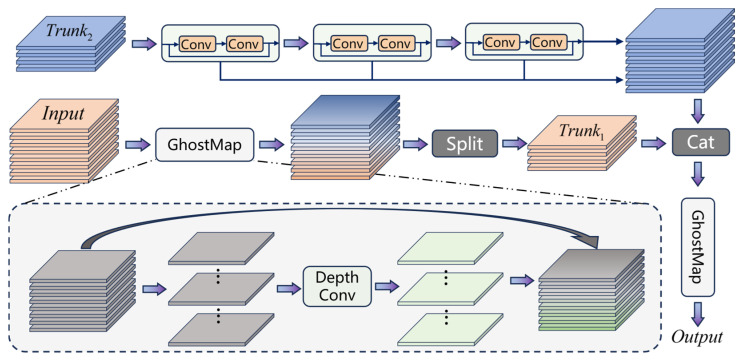
Architecture diagram of the Ghost Feature Bottle Cross module (GFBC) structure.

In the model’s pyramid backbone, as feature layers are propagated upwards, the number of channels increases while the feature maps become smaller. However, in the lower levels of the pyramid, the semantic information is often of lower dimensionality. The original design of backbone modules, such as C2f [11,40], aims to ensure an adequate amount of information. Yet, when generating a large number of feature layers, this approach can lead to a significant computational burden due to extensive convolutional operations and simultaneously reduce the internal information utilization efficiency of the model. To address these issues, we propose the use of Ghost Convolutions to alleviate the heavy computational load of high-density convolutions within the module [14], as shown in Figure 5. The advantage of Ghost Convolutions lies in their ability to generate a large number of semantically similar convolutional features based on computations involving only a small number of channels. This characteristic ensures consistent feature representation in convolutional modules with low-dimensional semantics. Moreover, to accommodate variations in object scale within infrared images, the module requires additional scale semantics. This necessitates providing unequal receptive fields across different convolutional layers to adapt to the features effectively.

The proposed module is named the Ghost Feature Bottle Cross module (GFBC), which retains the Bottle Cross property found in CSP (Cross Stage Partial) structures [12]. Building upon this foundation, we have enhanced the feature hiding capacity of the bottleneck, thereby increasing the feature compression rate. Additionally, the incorporation of Ghost features allows for a longer convolution channel, enabling a greater number of channels under similar computational costs. In the initial convolution layer, we implement Ghost convolutions using a C1 channel count that matches the size of the input layer. Specifically, half of the convolution layers are derived from dense convolutions (expensive convolutions), while the other half are generated from depthwise separable convolutions (cheap convolutions) [41]:

(1) $$\left\{\begin{array}{l} f^{E}=Norm(Conv(f^{In}))\\ f^{C}=Norm(DepConv(f^{E})) \end{array}\right.$$

We implement the expensive convolutions using a basic 3 × 3 convolution kernel and the depthwise convolutions using a larger 5 × 5 kernel for the other half. The 5 × 5 depthwise convolutions can cover a substantial receptive field with relatively low computational cost, thereby reinforcing the basic features further.

The combination of Ghost features and the Bottle Cross structure achieves good compatibility. This is because the two groups of receptive fields in the Ghost features are not identical, which provides a basis for the Cross mechanism. We retain one group of Ghost features and process the other group through multiple levels of Bottle operations, where all convolutional kernels are 3 × 3. As a result, the receptive field gradually increases, building upon a 5 × 5 receptive field:

(2) $$f^{BO}=[f^{E},f^{C},f^{B1},f^{B2},f^{B3}]$$

where $f^{BO}$ denotes the merged output of the Bottle Cross structure, which is composed of multiple concatenated convolutional features. Therefore, within modules of the same size, we integrate six distinct receptive fields, significantly enhancing the detection of objects of varying sizes. At the end of the model, we continue to use the GhostMap to fuse multiple feature groups, simultaneously constructing the other half of the features at a low computational cost. This feature distribution will be carried over to the next layer.

### 4.3. Scale Feature Recombination Module

Feature extraction by the model’s backbone is not the endpoint of image information processing. For tasks such as object recognition, to further reduce computational cost and improve accuracy, feature filtering is performed in the neck layer. Therefore, the feature utilization efficiency of the neck layer directly determines the accuracy of the model’s output. The features provided to the head layer are all compressed by the neck layer, which was somewhat limited by the original Base Conv Bottleneck module in the baseline algorithms. To address this, we propose a new module with higher information density.

**Figure 6:**
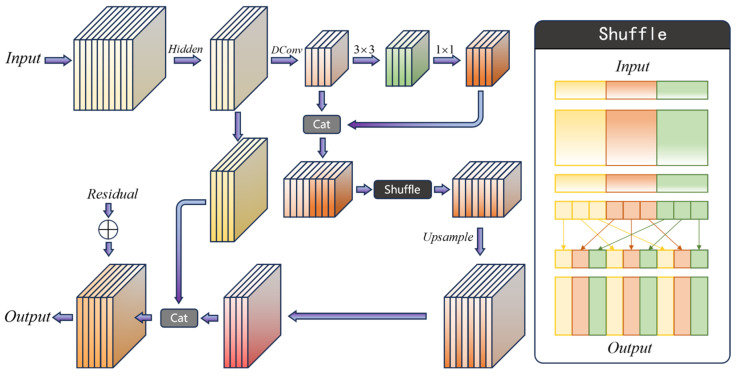
Architecture diagram of the Scale Feature Recombination module (SFR) structure.

We propose a new feature selection module for the neck layer called the Scale Feature Recombination module (SFR), as shown in Figure 6. The core of the SFR module lies in a series of downsampling and upsampling transformations followed by channel shuffling [17]. Compared to directly increasing the scale, shuffling the high-density tensor information facilitates a better combination with the original features during upsampling.

In the SFR module, a hidden layer is implemented using half the number of input channels. During downsampling, downsample Conv is used to achieve better gradient propagation, and the features extracted from the front and rear ends are merged and shuffled. Let *C* denote the number of channels and *G* the number of shuffle groups; the original feature group set is

(3) $$\left\{\begin{array}{l} M=C/G\\ S_{i}^{f}=\{f_{j}\},i\in 1,2,\ldots ,G,j\in M(i-1),\ldots ,Mi \end{array}\right.$$

where $S_{i}^{f}$ denotes the feature set of the *i*-th group, and $f_{j}$ represents the *j*-th channel within the batch. Then, the channels within each feature group are shuffled:

(4) $$Swap(S_{i,j}^{f},S_{j,i}^{f}),i\in 1,2,\ldots ,G,j\in i+1,\ldots ,G,i\neq j$$

where $S_{i,j}^{f}$ denotes the *j*-th feature of the *i*-th set. At this point, the input tensor features have been recombined through individual channel shuffling, resulting in the concatenation of all sets, where $f^{O}$ represents the output tensor:

(5) $$f^{O}=[S_{1}^{f},S_{2}^{f},\ldots ,S_{G}^{f}]$$

Finally, we merge the shuffled features with the original-sized features to ensure compatibility with different sampling rates.

### 4.4. Mask Comprehensive Loss

The baseline algorithm for box recognition has undergone numerous iterations, incorporating Class BCE Loss, Box CIoU Loss, and Distribution Focal Loss [40]. However, the design of the Mask Loss is insufficiently comprehensive. In the baseline algorithm, the ground truth region is used to compute the BCE loss, which, although covering all pixels within the region, lacks sufficient accuracy in guiding the convergence of segmentation masks.

To address this issue, we propose the Mask Comprehensive Loss, which includes two improvement terms: Mask Weighted BCE Loss and Mask Dice Loss [19]. These enhancements aim to improve the accuracy and robustness of mask segmentation.

#### 4.4.1. Mask Weighted BCE Loss

Binary Cross-Entropy (BCE) loss is the most classical binary classification loss, designed for general scenarios where the target occupies a significant portion of the image, leading to a relatively balanced distribution of foreground and background. However, in the context of drone perspectives, our primary focus is on vehicles, which occupy a much smaller area in the image. As shown in Figure 3d, the ratio of the contour area to the Box area differs significantly from the balanced midpoint.

Based on these observations, we propose the Weighted BCE Loss to address the issue of pixel imbalance in recognition tasks:

1. When the Box is significantly different from the ground truth, the number of negative sample pixels far exceeds that of positive samples, leading to local mask loss failure.
2. When the Box is close to the ground truth, the loss should focus on rapidly fitting the target pixels, but the standard BCE loss tends to lose focus.

The Weighted BCE Loss applies weighting to two regions: the foreground and the background. We achieve this using two hyperparameters, α and β.

(6) $$WeightMap=\alpha W^{F}+\beta W^{B}$$

where $W^{F}$ represents the foreground weight map, and $W^{B}$ represents the background weight map. These two maps are combined to form the BCE weight map. Therefore, the combined loss is

(7) $$L_{BCE}^{MW}=WeightMap\times L_{BCE}$$

where $L_{BCE}^{MW}$ represents the Weighted BCE Loss.

#### 4.4.2. Mask Dice Loss

Binary Cross-Entropy (BCE) loss guides each pixel in the predicted region to approach the ground truth, but the convergence speed in this scenario is not ideal. This is because the overall convergence of the mask towards the ground truth is too slow, especially for the positive pixels. In object detection, overall convergence is typically achieved using IoU Loss, which requires specifying another hyperparameter, the box score threshold. However, in mask segmentation, it is not feasible to explicitly set a 0–1 threshold for the mask, as low-score mask pixels are crucial for fitting the data during the early stages of model training. If these pixels are filtered out due to the threshold, it could have a counterproductive effect. Therefore, to ensure the overall convergence of the mask while avoiding the loss of positive pixels, we incorporate Mask Dice Loss as an additional term in the loss function.

Similar to IoU, Dice Loss considers the intersection and union in its computation, but it does not require the introduction of additional hyperparameters.

(8) $$Dice=2\times \frac{M^{P}\cap M^{GT}}{\left|M^{P}\right|+\left|M^{GT}\right|}$$

In this scenario, any positive values within the mask prediction region are included in the computation, allowing low-score regions to quickly approach the ground truth region rather than being ignored. Additionally, we introduce a weight coefficient η for the Dice loss, ensuring that it complements the BCE loss without overwhelming it.

(9) $$L_{MD}=\eta (1-Dice)$$

where $L_{MD}$ represents the Mask Dice Loss. Therefore, the final total loss function constructed by our method is

(10) $$Loss=L_{cls}+L_{CIoU}+L_{DF}+L_{BCE}^{MW}+L_{MD}$$

## 5. Experiments

We trained and tested our algorithm on the DIVIS dataset, which was divided into a training set and a validation set in a 4:1 ratio. The algorithm was implemented on a platform equipped with an Intel(R) CPU i7-13790F and a Nvidia RTX 3090 GPU. Our core hyperparameter settings are as follows: Mask BCE Weight α = 1.2, β = 0.85, and Mask Dice weight η = 0.2. The model was trained for a total of 100 epochs with a batch size of 32.

### 5.1. Contrast Experimtes

Our research focuses on real-time instance segmentation algorithms, which primarily consider models with low computational requirements. In the experimental phase, we used lightweight backbone models with minimal parameters for comparison. The methods include classic instance segmentation algorithms such as BoxInst-R18 [26], SOLOv2-R18 [29], RTMSeg-CSPTiny [30], YOLOACT-D19 [31], CondInst-R18 [24], YOLOv8n-Seg [11], YOLOv11n-Seg [42], and MonoSeg (proposed). Here, R18 indicates that the backbone network uses the ResNet18 architecture [13], CSPTiny denotes the use of the smallest CSP backbone [15], D19 signifies the use of the DarkNet19 backbone [43], and YOLOn represents the nano-sized YOLO model structure. Despite using the same or similar parameter-count backbones, these algorithms differ in their neck and head structures, leading to significant variations in computational complexity and processing time.

Considering the inherent speed disadvantages of two-stage algorithms such as Mask R-CNN [18], we do not include two-stage image segmentation algorithms in the speed comparison. Instead, we focus solely on single-stage image segmentation algorithms to ensure that the computational complexities of the compared methods are similar, thereby providing a more comprehensive performance comparison.

We organized the output results of all the algorithms into COCO format and used the COCOTools library to uniformly compute the precision metrics. The model parameters and computational complexity were calculated using the ThOP (Torch Operations Profiler) tool. The frames per second (FPS) were measured separately for each algorithm on a unified testing platform.

#### 5.1.1. Basic Performance Comparison

The primary accuracy and speed metrics focus on the basic mAP (mean Average Precision) for both Box and Mask, as well as the FPS. For high-speed recognition models, temporal performance is a core metric.

As indicated in Table 2, MonoSeg achieves a satisfactory balance between model accuracy and computational efficiency. Although the precision of Box0.5 and Mask0.5 is not as high as that of CondInst and RTMSeg algorithms, MonoSeg’s accuracy surpasses that of two algorithms from the YOLO family, showcasing its good adaptability to various scenarios. BoxInst, being a weakly supervised instance segmentation algorithm, also demonstrates a high level of precision performance. In contrast, classic algorithms such as YOLACT and SOLOv2 exhibit suboptimal performance on the DIVIS dataset.

However, the aforementioned algorithms exhibit significant differences in terms of parameter count and inference speed. The BoxInst weakly supervised algorithm, for instance, consumes a substantial amount of computational resources to integrate semantically similar pixels for segmentation, with a computational load reaching 34.9 G and FPS as low as 19. Although CondInst demonstrates excellent accuracy, it also has a high computational demand and slower inference speed. In contrast, SOLOv2 and YOLACT offer faster performance, achieving 45 FPS and 67 FPS, respectively. Nevertheless, these algorithms are still relatively slow for embedded engineering deployment. On the other hand, the YOLO series of algorithms and MonoSeg show considerable advantages. Thanks to their efficient low-computation model architectures, all models have a parameter count not exceeding 10 M, and they achieve a FPS greater than 100. Among them, the YOLOv11 algorithm outperforms MonoSeg in terms of inference speed, primarily due to its high parameter reuse rate and parallel inference capabilities, which contribute to the lowest computational load among the evaluated models.

**Figure 7:**
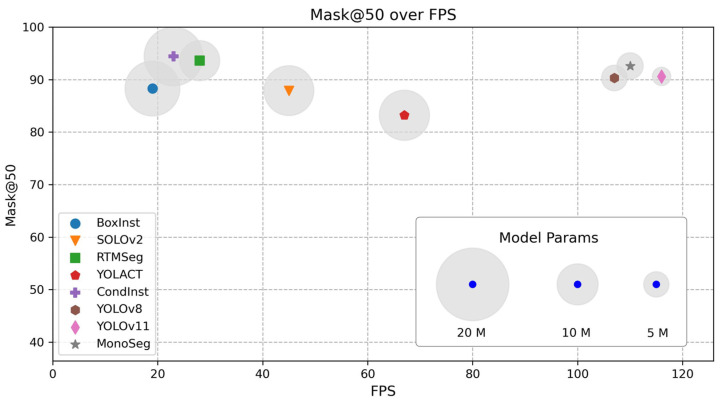
Scatter plot of model inference frame rate vs. Mask0.5 mAP.

As shown in Figure 7, MonoSeg outperforms the baseline models in both accuracy and speed, achieving a well-balanced trade-off between the two, which endows it with considerable engineering value. Compared to multi-branch structured algorithms such as CondInst and RTMSeg, MonoSeg exhibits a significant speed advantage. When compared to YOLOv8 and YOLOv11, MonoSeg also shows a certain advantage in accuracy while maintaining nearly equivalent frame rates. Therefore, the algorithm proposed in this paper possesses engineering advantages. Meanwhile, MonoSeg exhibits a notable advantage over several other methods in terms of the number of parameters.

#### 5.1.2. Multiple Threshold Performance Comparison

By comparing the model performance under multiple threshold conditions, this study analyzes the balance of the algorithms. Specifically, the performance of eight algorithms is evaluated using the Box0.75, Box0.5–0.95, Mask0.75, and Mask0.5–0.95 criteria. High IoU (Intersection over Union) threshold metrics reveal the extreme performance capabilities of the algorithms. The detailed results are illustrated in the following figure.

**Figure 8:**
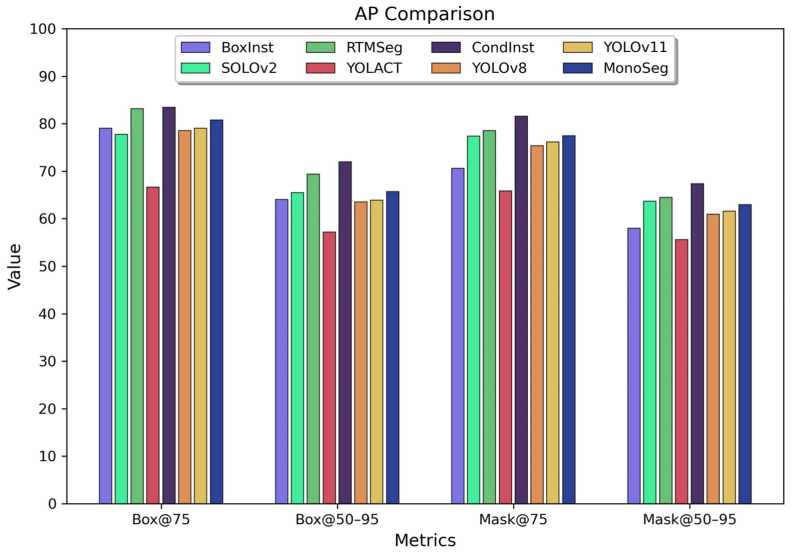
Bar chart comparing Box and Mask mAP across multiple thresholds.

As shown in Figure 8, after comparisons across multiple thresholds, CondInst and RTMSeg exhibit the best overall performance at high IoU thresholds, benefiting from their high-precision Mask segmentation methods without ROI filtering. MonoSeg demonstrates relatively balanced characteristics, whereas BoxInst shows greater fluctuations in both Box and Mask precision. SOLOv2 also stands out with its Mask precision at high thresholds. The YOLOv8 and YOLOv11 baseline algorithms display a step-like characteristic, further highlighting the performance improvement achieved by the MonoSeg algorithm.

As presented in Table 3, we provide a comparative analysis of the mAP0.5–0.95 recognition performance for objects categorized into small, medium, and large size threshold categories. By stratifying the targets based on their pixel area into three tiers, we evaluate the algorithm’s recognition versatility across these categories. Under the premise of maintaining high absolute performance, closer values among the three metrics indicate a more balanced algorithm performance, which, in turn, signifies a stronger adaptability to diverse scenarios. The MonoSeg algorithm demonstrates a well-balanced profile across all three size categories, notably surpassing all other algorithms in the recognition of large objects. This evidence underscores MonoSeg’s superior performance across all evaluated scenarios.

#### 5.1.3. Multiple Class Performance Balance Comparison

The DIVIS dataset categorizes target instances into two classes. To investigate the recognition capabilities of different algorithms on distinct object categories, metrics for various algorithms are separately calculated for the two categories. The metric used is AP0.5, where B/MN0.5 denotes the AP0.5 precision of Box or Mask for the N-th class.

As shown in Table 4, CondInst, MonoSeg, YOLOv11, and SOLOv2 all exhibit outstanding performance across various metrics. However, exceptional performance in a single category does not necessarily reflect the overall capability of an algorithm but rather indicates a design bias. CondInst, for instance, significantly outperforms other algorithms in the accuracy of small vehicle targets, yet its performance gap in recognizing large vehicle targets is substantial, leading to an imbalance in overall performance. In contrast, MonoSeg, SOLOv2, and the two YOLO algorithms show more balanced performance, with high recognition rates for both small and large targets. Therefore, a comprehensive comparison confirms that the MonoSeg algorithm possesses a comprehensive advantage across multiple performance indicators.

**Figure 9:**
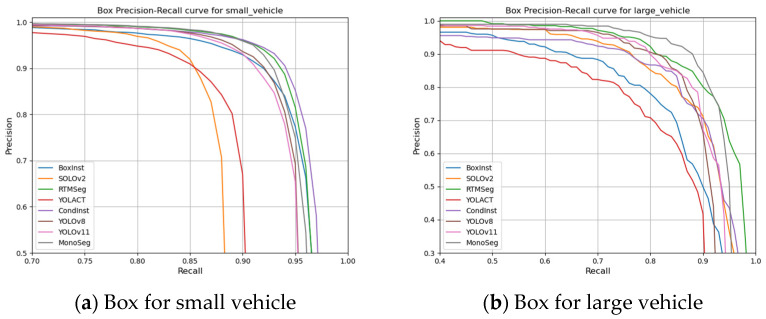

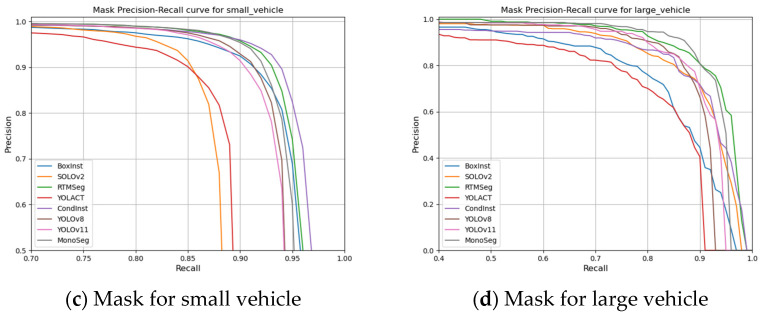
Two classes of target Box and Mask P–R curves.

As shown in Figure 9, we present the Precision–Recall curves for Box and Mask at the IoU threshold of 0.5 for multiple algorithms. The comparison reveals that the average precision for large vehicles is somewhat lower than that for small vehicles, with greater variability and a wider fluctuation range, indicating less stability. Notably, algorithms such as CondInst and BoxInst exhibit higher accuracy in detecting small vehicles but experience a significant drop in performance when identifying large vehicles. Conversely, SOLOv2 shows the opposite trend, with better performance on large vehicles. In contrast, the MonoSeg algorithm demonstrates consistent precision across both categories of targets. This analysis highlights the varying effectiveness of different algorithms in handling objects of different sizes. The MonoSeg algorithm’s stable performance underscores its robustness and adaptability to diverse target dimensions, making it particularly suitable for scenarios where both small and large vehicles are present.

### 5.2. Abalation Study

An ablation study is conducted to analyze the three proposed improvements in this paper, delving into the effects of each module and the enhancements to the loss function. Here, we collectively refer to the two proposed Mask Loss as Comprehensive Loss (C-Loss). The evaluation metrics used include Box0.5, Box0.75, Mask0.5, and Mask0.75. Specifically, the AP0.5 metric serves as an indicator of the model’s basic recognition capability, while AP0.75 is considered an advanced metric.

As shown in Table 5, we compare three improvements against the Baseline. When the GFBC module is introduced, the model’s Box0.5 performance increases by approximately 0.7%, and the Mask0.5 performance improves by about 0.6%. Additionally, there is a notable enhancement in the model’s AP0.75 performance, which validates the positive impact of our proposed GFBC module within the backbone. Moreover, due to the feature cheap reconstruction capability of the GFBC module, the model’s computational cost decreases by 0.6 GFLOPs, leading to a further improvement in inference speed.

Building upon the Baseline + GFBC module, we further introduce the SFR module. With this addition, the model’s Box0.5 performance improves by 1.3% compared to using the GFBC module alone, and the Mask0.5 performance increases by 1.5%, both surpassing the results obtained with the GFBC module alone. The combination of GFBC and SFR yields a 1.9% improvement in Box0.5 performance and a 2.2% improvement in Mask0.5 performance relative to the baseline approach. Although the model’s performance sees a significant boost, the introduction of the SFR module also leads to an increase in computational cost by 0.2 GFLOPs, thereby adding to the model’s computational burden.

Finally, we introduce the C-Loss improvement, which is specifically designed to enhance the convergence of the Mask. While this improvement also positively affects the Box recognition accuracy, the increase in Box precision is not substantial; the Box0.5 performance improves by only 0.4% compared to the previous stage. However, the Mask0.5 precision sees a significant boost, increasing by approximately 1.0% relative to the previous stage. Importantly, the improvement in the loss function does not add any additional computational cost, offering an advantage for the overall optimization of the model.

In summary, our experiments demonstrate that each of the three improvements contributes to performance enhancements, albeit with different focal points and degrees of improvement. Collectively, these enhancements form the foundation of our MonoSeg algorithm.

### 5.3. Visualization Experiments

To provide a more intuitive comparison of the recognition performance between the proposed algorithm and other algorithms, the prediction results of the proposed algorithm and the comparative algorithms are visualized. In this visualization, the confidence threshold for instance predictions is set to 0.45, and the visualization format is uniformly presented as bounding boxes + segmentation masks + central confidence scores.

**Figure 10:**
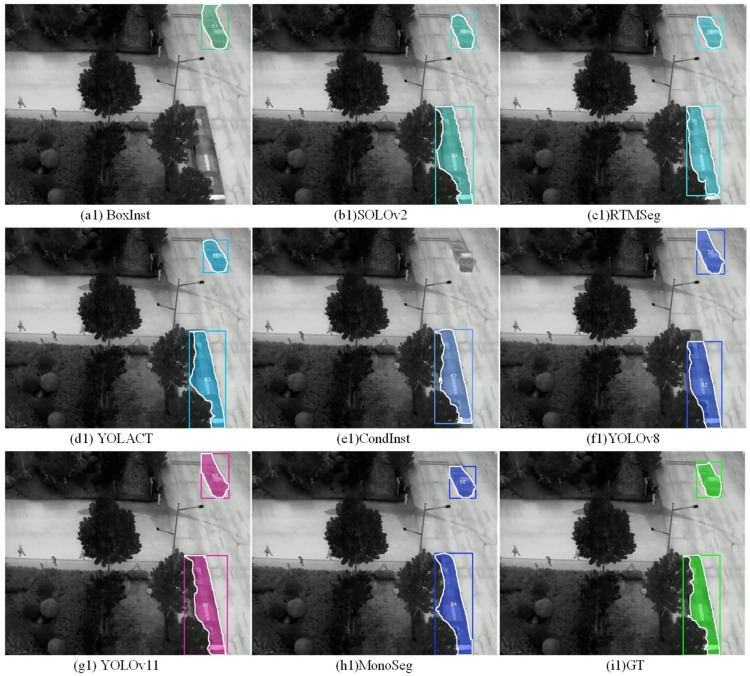

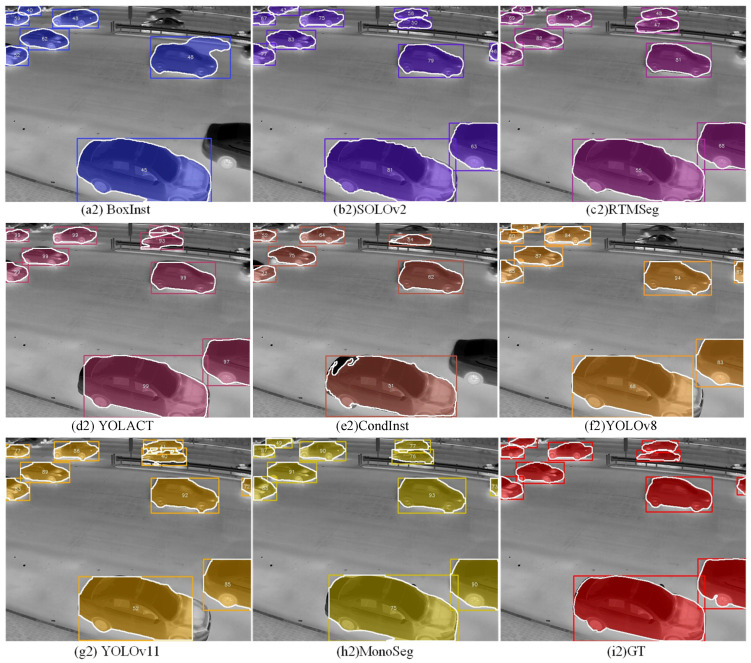
Visualization examples of comparative experimental results. (**1**) and (**2**) represent the segmentation results of the two images respectively, (**a**–**h**) represents each method, and (**i**) represents GT. Red indicates a small vehicle and green indicates a large vehicle.

As shown in the recognition results from Figure 10, the proposed MonoSeg algorithm exhibits a certain advantage in recognition accuracy. Algorithms like CondInst, BoxInst, and RTMSeg use high-accuracy segmentation heads, which gives them an edge in fine-grained segmentation compared to YOLO and MonoSeg. However, in image example (1), BoxInst and CondInst show instances of missed detections, while RTMSeg displays incomplete segmentation. Similarly, in image example (2), these issues are evident again, and additionally, the YOLACT, SOLOv2, and YOLOv11 algorithms also exhibit problems with incomplete segmentation. In contrast, MonoSeg provides more complete and balanced recognition of multiple targets.

To intuitively compare the effectiveness of target feature regions, this paper visualizes the heatmaps of the seventh layer of the backbone for both the baseline algorithm and MonoSeg. The seventh layer of the backbone corresponds to the third level of the feature pyramid structure, representing a feature space where the scope of feature action is neither too broad nor too narrow. This layer effectively reflects the intensity of feature mappings at different scales.

**Figure 11:**
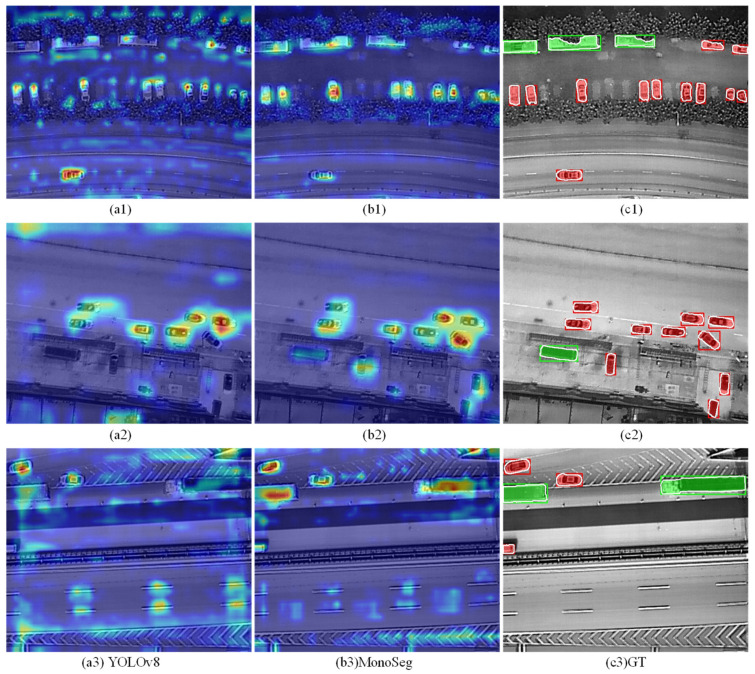
Visualization comparison of heatmaps between baseline and MonoSeg algorithms. (**1**–**3**) represents the segmentation results of the three images respectively; (**a**,**b**) represent the heatmap of YOLOv8 and MonoSeg respectively; (**c**) represents GT. Red indicates a small vehicle and green indicates a large vehicle.

Comparing the heatmap visualizations in Figure 11, MonoSeg demonstrates superior focus and coverage of hot regions compared to the baseline algorithm. As shown in image (1) and image (2), the heatmap information from MonoSeg can largely cover all targets, whereas the baseline algorithm exhibits some omissions. In image (3), background interference impacts the recognition performance; the baseline algorithm shows a notably higher attention to the background crosswalk areas compared to MonoSeg. This evidence supports the conclusion that the proposed algorithm has better foreground–background discrimination capabilities than the baseline algorithm.

## 6. Discussion

We have designed the DIVIS dataset and the MonoSeg instance segmentation algorithm for the task of vehicle target instance segmentation in infrared images captured from a drone perspective. The DIVIS dataset is constructed based on two categories of vehicle targets, which exhibit significant differences in size characteristics. This presents both challenges and opportunities for our algorithm design. To address the issues of varied image features, imaging blurriness, and extreme target sizes, we propose the following improvements:

1. A multi-level receptive field backbone model with low computational cost.
2. An efficient information filtering neck under multi-feature scale conditions.
3. Loss function optimization for addressing class imbalance between positive and negative samples.

Therefore, we propose the MonoSeg algorithm, which is an improvement upon the YOLOv8-Seg baseline algorithm. MonoSeg introduces the Ghost Feature Bottle Cross module (GFBC), the Scale Feature Recombination module (SFR), and Comprehensive Loss optimization. On the DIVIS dataset, MonoSeg achieves a 2.4% improvement in mAP Box0.5 and a 2.5% improvement in mAP Mask0.5. Additionally, the model’s computational cost is reduced by 0.4 GFLOPs while maintaining a balance between inference speed and accuracy. This enables high-precision instance segmentation recognition under high-speed prediction conditions.

However, the MonoSeg algorithm also reveals certain limitations. For instance, by inheriting the prototype from YOLO and YOLACT segmentation algorithms, it faces challenges in ensuring fine-grained pixel-level segmentation. Future work aims to achieve more refined segmentation outcomes through the integration of low-level pixel semantics with high-level prototypes. Moreover, its reliance on a fixed number of anchors limits its effectiveness in scenarios with extremely dense targets. To address this issue, future developments will focus on adaptively adjusting the number of anchors based on the algorithm, allowing the model’s head layer to make self-adaptive decisions regarding anchor density. This approach seeks to enhance the effective recognition of densely packed, small-sized objects.

Our future research will focus on advancing this field through several key initiatives. These include expanding datasets to encompass a broader spectrum of scenarios and objects, as well as deeply optimizing the algorithm design. Such efforts will lay the foundation for developing algorithms with enhanced precision and speed. We also anticipate that these advancements will enable our algorithms to adapt to more complex environments and application domains, such as fire early warning systems and wildlife monitoring, thereby broadening their applicability. Ultimately, our goal is to deploy and optimize these general-purpose algorithms on embedded systems within UAV, facilitating genuine edge device applications.

## 7. Conclusions

This study proposes MonoSeg, an instance segmentation algorithm with strong adaptability and integrity. Specifically designed for the task of infrared vehicle target segmentation from a drone perspective, we introduce the Ghost Feature Bottle Cross module (GFBC), the Scale Feature Recombination module (SFR), and Comprehensive Loss improvements, building upon the YOLOv8n-Seg algorithm. The proposed algorithm was tested and validated on the DIVIS dataset constructed for this study. Compared to the baseline algorithm, MonoSeg achieves a balanced improvement in both performance and speed, making it an ideal algorithm for deployment on embedded devices.

## Figures and Tables

**Table 1:** Properties of the four image datasets and the number of images sampled.

Number	Dataset Name	Main Scene	Camera Angle	Original Count	Sampled Count
1	Aerial-Mancar [37]	Street, Buildings	<60°	11,045	1136
2	VisDrone [38]	Street, Bridge	30–90°	28,439	1645
3	HIT-UAV [39]	Square, Street	30–90°	2898	195
4	Self-collected	Treelawn, Street	45–90°	3955	327

**Table 2:** Table of basic performance comparison.

Method	Params	Box0.5	Mask0.5	FLOPs	FPS
BoxInst-R18 [26]	14.4 M	89.3	88.3	34.9 G	19
SOLOv2-R18 [29]	12.8 M	87.9	87.9	20.9 G	45
RTMSeg-CSPTiny [30]	9.7 M	94.2	93.6	27.5 G	28
YOLACT-D19 [31]	12.9 M	83.7	83.2	17.4 G	67
CondInst-R18 [24]	15.6 M	92.5	94.4	33.8 G	23
YOLOv8n-Seg [11]	5.1 M	90.7	90.3	12.1 G	107
YOLOv11n-Seg [42]	2.8 M	91.3	90.6	10.4 G	116
MonoSeg (Proposed)	5.3 M	92.9	92.6	11.7 G	110

**Table 3:** Table of three-level area mAP0.5–0.95 performance comparison.

Method	Box^S^	Box^M^	Box^L^	Mask^S^	Mask^M^	Mask^L^
BoxInst-R18 [26]	54.1	70.6	60.6	45.9	61.7	52.0
SOLOv2-R18 [29]	43.1	71.8	81.4	41.9	70.1	75.4
RTMSeg-CSPTiny [30]	53.2	72.6	76.9	46.4	70.1	72.5
YOLACT-D19 [31]	39.3	63.4	64.7	36.3	61.8	63.8
CondInst-R18 [24]	57.8	68.9	78.6	51.0	72.7	73.4
YOLOv8n-Seg [11]	44.8	69.2	77.5	41.2	67.2	75.4
YOLOv11n-Seg [42]	47.0	69.0	75.1	42.2	66.5	72.8
MonoSeg (Proposed)	49.1	71.2	82.3	43.9	69.0	77.9

**Table 4:** Table of multi-class performance comparison.

Method	B1AP0.5	B2AP0.5	M1AP0.5	M2AP0.5
BoxInst-R18 [26]	94.4	84.1	93.6	82.9
SOLOv2-R18 [29]	86.9	89.1	86.7	89.0
RTMSeg-CSPTiny [30]	94.2	92.1	93.6	92.6
YOLACT-D19 [31]	87.9	79.5	86.9	79.3
CondInst-R18 [24]	95.5	87.6	95.2	87.7
YOLOv8n-Seg [11]	93.9	88.4	92.9	88.3
YOLOv11n-Seg [42]	94.0	88.9	93.3	89.1
MonoSeg (Proposed)	94.8	91.4	93.7	91.4

**Table 5:** Table of ablation study performance comparison.

Method	Box0.5	Box0.75	Mask0.5	Mask0.75	FLOPs
Baseline	90.7	78.6	90.3	75.4	12.1 G
+GFBC	91.3	79.1	90.9	76.0	11.5 G
+GFBC+SFR	92.5	80.4	91.7	77.1	11.7 G
+GFBC+SFR+C-Loss	92.9	80.8	92.6	77.5	11.7 G

## Data Availability

The data presented in this study are available upon request from the author.
